# Extrapancreatic solid pseudopapillary neoplasm: Report of a case of primary retroperitoneal origin and review of the literature

**DOI:** 10.3892/ol.2013.1242

**Published:** 2013-03-11

**Authors:** HEJIA ZHU, DAN XIA, BO WANG, HONGZHOU MENG

**Affiliations:** 1 Departments of Urology, Hangzhou, Zhejiang 310003, P.R. China; 2 Pathology, The First Affiliated Hospital of Zhejiang University, Hangzhou, Zhejiang 310003, P.R. China

**Keywords:** solid pseudopapillary tumors, ectopic pancreas, retroperitoneum, origin

## Abstract

Solid pseudopapillary tumors (SPTs) occurring as primary tumors outside the pancreas are exceedingly rare. The present study reports such a case occurring as a non-functional adrenal tumor in a 22-year-old female. The tumor was completely removed from the retroperitoneum by laparoscopic surgery. A well-defined, encapsulated tumor measuring 6×6×5 cm was histologically characterized by a combination of the solid and pseudopapillary growth patterns of tumor cells with eosinophilic cytoplasm. Ectopic pancreatic tissue was also found histologically within the resected tumor. On immunostaining, the tumor was positive for progesterone receptor, CD56, cytokeratin and CD10. The morphological and immunohistochemical features were compatible with those of SPT. To the best of our knowledge, this is the first case report of extrapancreatic SPT with evidence of a pre-existing ectopic pancreas in the retroperitoneum. A review of the published English literature uncovered 12 cases of extrapancreatic SPTs, and revealed that extrapancreatic SPTs are likely to have a favorable clinical course and a clinical profile similar to their pancreatic counterparts.

## Introduction

A solid pseudopapillary tumor of the pancreas (SPTP) is an uncommon pancreatic neoplasm with low malignancy that accounts for 0.1–3% of all exocrine pancreatic tumors, with marked adolescent female predominance. Since first described by Frantz in 1959 (1), there have been >700 well-documented cases of SPTP reported in the published English literature (2). However, SPTs that occur as primary tumors outside the pancreas are exceedingly rare. The most common extrapancreatic site is the mesocolon or omentum, where the tumor arises in ectopic pancreatic tissue (3–6). Recently, Miyazaki *et al* reported one case of SPT without an ectopic pancreas in the retroperitoneum, a site not previously recognized to harbor this type of tumor (7). The present study reports an additional case of this rare tumor located in the retroperitoneum and the histological observations of a pancreatic rest. A detailed review of the literature concerning extrapancreatic STPs is also discussed. Written informed consent was obtained from the patient.

## Case report

**Figure 1:**
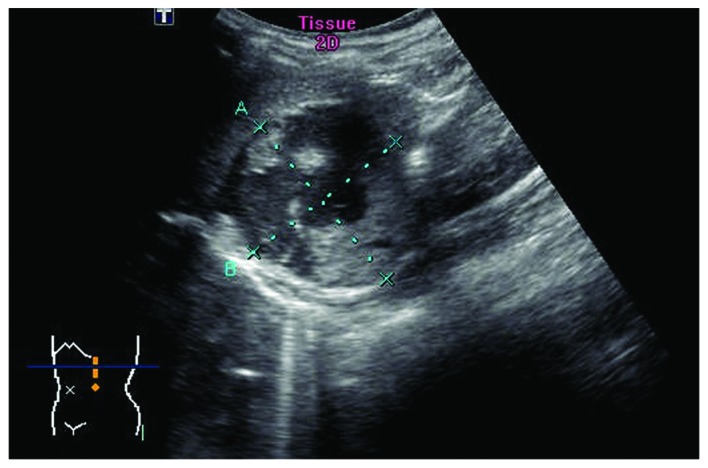
B ultrasound imaging showing a 5.9×5.3-cm retroperitoneal mass in the left adrenal region.

**Figure 2:**
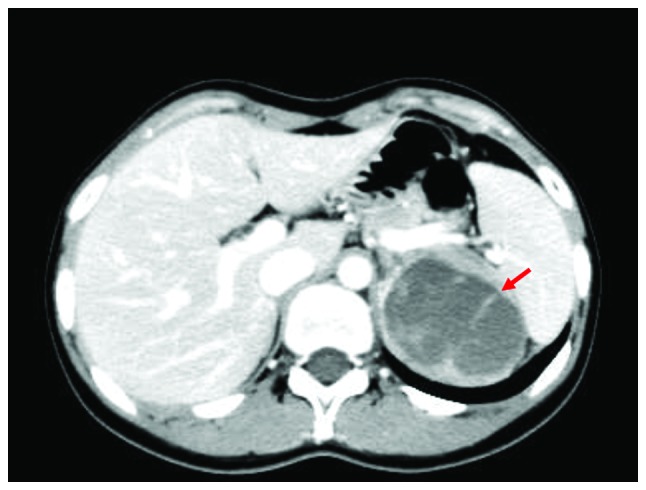
Computed tomography scan showing an adrenal mass suspected to be a possible adrenal tumor.

A 22-year-old female was referred to the First Affiliated Hospital of Zhejiang University School of Medicine, Zheijang, China, due to the presence of a retroperitoneal 5.9×5.3-cm mass in the left adrenal region that was detected by B ultrasound (BUS) imaging during a routine annual examination (Fig. 1). The patient did not complain of fatigue, fever or pain, and the patient’s body weight and blood pressure were within the corresponding normal ranges. The complete blood count, chemistry panel, urine analysis and liver function test results were also normal. An endocrinological evaluation of the adrenal gland, including an analysis of the plasma rennin activity and aldosterone, steroid and catecholamine levels, excluded a functional adenoma. Abdominal computed tomography (CT) revealed that the retroperitoneal mass was solid, encapsulated and ∼6 cm in diameter, with low attenuation and containing solid and cystic components. The mass was located in the adrenal region; positioned close to the pancreatic tail, behind the stomach and between the spleen and the aorta (Fig. 2). Since the diagnosis of an adrenal tumor was not excludable based on the results of the BUS and CT examinations, the patient was scheduled for laparoscopic adrenalectomy. During the surgery, the pancreas and left adrenal gland were in contact and clearly separated from the tumor. The main blood supplies originated from branches of the splenic artery. The tumor was completely removed from the retroperitoneum during this laparoscopy.

**Figure 3:**
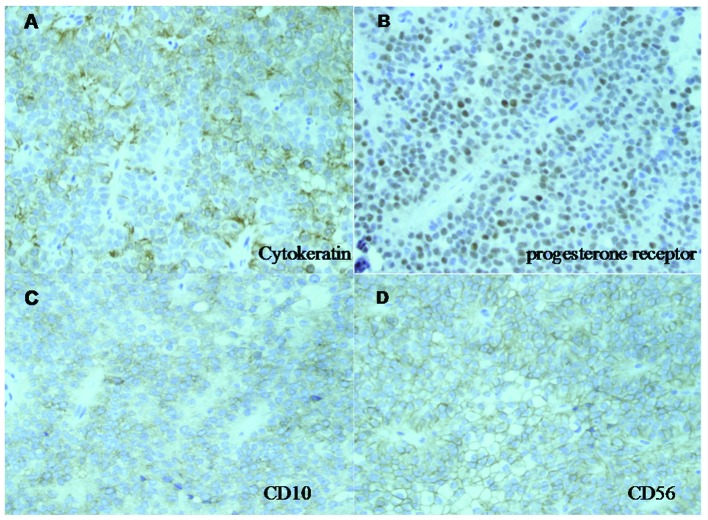
Biopsy of the tumor. Immunostaining for (B) the progesterone receptor and (D) CD56 is positive. Cytokeratin (A) and CD10 (C) are focally positive (×400).

Upon gross examination, the tumor was measured as 6×6×5 cm in size, with a wight of 80 g. The external surface was smooth, and the cut surfaces showed that the solid tumor was interspersed with cystic and necrotic spaces. Microscopically, the tumor was traversed by fibrovascular septa, forming broad-to-delicate pseudopapillae and hemorrhagic foci. The cores of certain pseudopapillae had a myxohyaline appearance. The tumor cells were cuboidal with round nuclei, finely granular chromatin and an eosinophilic cytoplasm. Mitotic figures were not observed. Immunostaining for the progesterone receptor and CD56 was positive. Cytokeratin and CD10 were focally positive. Staining for CD34, synaptophysin, α-1-antitrypsin and vimentin was negative (Fig.3). Ectopic pancreatic tissue was also observed histologically within the resected tumor. Based on these observations, SPT arising in a pancreatic rest of the retroperitoneum was diagnosed. At present, 14 months subsequent to the surgery, the patient is healthy without evidence of recurrence of SPT.

## Discussion

SPT is a relatively rare neoplasm, which is usually found in the body or tail of the pancreas. It has been described using various terms, including solid and cystic tumor, papillary epithelial neoplasm, papillary cystic neoplasm and Frantz’s tumor. In 2000, the World Health Organization classified this clinical entity as a ‘solid pseudopapillary tumor’ of the pancreas and further defined malignant SPT as present when tumor cells show angio-invasion, perineural invasion or deep invasion into the surrounding pancreatic parenchyma (8). The tumor may present at all ages and is observed to occur more often in Asian and African-American adolescent females (9). The mean age at diagnosis of the >700 cases reported to date is 28 years (range, 7–79 years), and 89% of the patients are female (10).

Tumors presenting as SPT but arising from extrapancreatic sites are extremely rare in the literature. A review of the English literature published since 1990 (using PUBMED) revealed only 12 cases of extrapancreatic SPT. These cases, along with that of the present study, are summarized in Table I. Among these 13 cases, there are 11 female patients and 2 male patients, with a mean age of 32 years (range, 13–73 years), who presented with large tumors (mean size, 8 cm) similar to the clinical profile of the pancreatic counterpart. The clinical presentation of extrapancreatic SPT is varied. Abdominal pain and a palpable mass were the most common symptoms; however, certain patients were asymptomatic and the tumor was incidentally detected. Notably, 3 out of the 13 cases involved tumors that arose from the ectopic pancreas, a well-developed and normally organised pancreatic tissue lying outside its normal location with no anatomical or vascular connections to the proper pancreas. Ectopic pancreases are reported to be detected at a frequency of 1–2% by autopsy. The most common sites for an ectopic pancreas are the stomach, 25–38%, the duodenum, 17–36%, and the jejunum, 15–22% (11). An ectopic pancreas may also be found in Meckel’s diverticulum, the biliary tract, the gallbladder, the liver and the spleen. To the best of our knowledge, the retroperitoneum is an extremely rare location for an ectopic pancreas and our case was the first report of extrapancreatic SPT with evidence of a pre-existing ectopic pancreas in the retroperitoneum.

The etiology of SPT is not well established. The immunohistological features of these tumors favor an epithelial origin, but certain cases of cytokeratin and vimentin coexpression raise the question of whether these tumors are of a mesenchymal nature (18). Increasing evidence supports the hypothesis that SPT may be associated with hormonally-sensitive tissues from the gonads. The exclusive occurrence of these tumors in young females, along with the positive presence of progesterone receptor markers, supports the theory that hormones have affects on the tumor development (19). Furthermore, the identification of extrapancreatic SPT in the ovaries also indicates a possible genital link, substantiated by the closeness of the genital ridges to the pancreatic anlage during embryogenesis (18). Thus, it is currently proposed that SPT originates from omnipotent cells associated with the genital ridges; a few of these cells may be entrapped in the pancreatic anlage during organogenesis, while others follow the normal migration pathway to the definitive location of the ovaries (15). According to this theory, extrapancreatic SPT may occur at any point along the route(s) followed by the migrating cells, including in the retroperitoneal space.

The clinical course of SPT is usually favorable as the tumors are indolent with <5% demonstrating aggressive behavior (20). Aggressiveness is generally correlated with cellular atypia, mitotic activity, prominent necrobiotic nests and invasion of the vascular spaces, perineural interstitium or neighbouring organs (21,22). Metastases occur with a low incidence of 14.7% and the majority are metastases of the liver (10). The optimal treatment for SPT remains as radical surgical resection, even in cases of metastatic disease, as the clinical progression following metastasis is slow and most lesions can be treated by complete surgical excision of the metastatic tumors (23). In patients with unresectable forms of the disease, there is limited evidence supporting the use of chemotherapy (24,25). Additionally, it is difficult to assess whether the long-term survival occasionally observed is attributable to the clinical benefits associated with chemotherapy, rather than the natural course of a poorly aggressive tumor. In the literature reviewed previously, metastasis was reported in 3 out of the 13 cases with extrapancreatic tumors; however, the majority of patients demonstrated long-term survival with no evidence of disease. The benign outcome is reassuring in that extrapancreatic STPs are likely to have a favorable clinical course similar to their pancreatic counterparts.

In conclusion, tumors presenting as SPT but arising from extrapancreatic sites are extremely rare. Certain SPTs may arise from the ectopic pancreas. The present study reported a case of this tumor type with a pancreatic rest that was located in the retroperitoneum. The etiology was also discussed. STPs are presently considered to originate from omnipotent cells associated with the genital ridges. A certain number of these cells may be entrapped in the pancreatic anlage to form SPTPs, while extrapancreatic SPTs may occur at any point along the route followed by the migrating cells during organogenesis, including the retroperitoneal space. The clinical course of SPT is usually favorable and extrapancreatic SPTs are likely to have a favorable clinical course that is similar to their pancreatic counterparts.

## Figures and Tables

**Table I:** Clinical features of reported cases of extrapancreatic solid pseudopapillary neoplasms.

Age (years)	Gender	Location	Size (cm)	Presentation	Ectopic pancreas	Therapy	Recurrence	Follow-up (months)	Outcome	Reference
13	F	Mesocolon	8	Abdominal pain	+	Open surgery	-	36+	Living/NED	3
15	F	Mesocolon	21	Abdominal pain	+	Open surgery	NA	NA	NA	5
46	F	Omentum	5	Asymptomatic	-	Laparotomy	-	3+	Living/NED	12
45	M	Omentum	18	Abdominal mass	-	Laparotomy	Liver and peritoneum	98	Succumbed to primary disease	13
31	F	Liver	30	Abdominal distention	-	Open surgery	-	13+	Living/NED	14
17	F	Left ovary	25.5	Abdominal mass	-	Open surgery	-	72+	Living/NED	15
57	F	Right ovary	3	Asymptomatic	-	Open surgery	NA	NA	NA	15
21	F	Left ovary	14	Abdominal swelling	-	Open surgery	NA	NA	NA	15
25	F	Right ovary	16.5	Abdominal fullness	-	Open surgery	-	144+	Living/NED	16
73	M	Duodenum	14	Vomiting, fatigue and weight loss	-	Open surgery	Liver	3	Succumbed	17
32	F	Stomach	10	Asymptomatic	-	Open surgery and chemotherapy	Liver, ovary and peritoneum	120+	Living	17
22	F	Retroperitoneum	8	Abdominal discomfort	-	Laparotomy	-	6+	Living/NED	7
22	F	Retroperitoneum	6	Asymptomatic	+	Laparotomy	-	14+	Living/NED	Present case

NED, no evidence of disease; F, female; M, male; NA, not available.

## References

[1] Frantz VK (1959). Tumors of the pancreas. Atlas of Tumor Pathology.

[2] Papavramidis T, Papavramidis S (2005). Solid pseudopapillary tumors of the pancreas: review of 718 patients reported in English literature. J Am Coll Surg.

[3] Ishikawa O, Ishiguro S, Ohhigashi H (1990). Solid and papillary neoplasm arising from an ectopic pancreas in the mesocolon. Am J Gastroenterol.

[4] Elorza Orúe JL, Ruiz Díaz I, Tubia Landaberea J, San Vicente Leza M (1991). Solid and papillary tumor on ectopic pancreas in transversal mesocolon. Rev Esp Enferm Dig.

[5] Tornóczky T, Kálmán E, Jáksó P (2001). Solid and papillary epithelial neoplasm arising in heterotopic pancreatic tissue of the mesocolon. J Clin Pathol.

[6] Kövári E, Járay B, Pulay I (1996). Papillary cystic neoplasms in the ectopic pancreas. Orv Hetil.

[7] Miyazaki Y, Miyajima A, Maeda T (2012). Extrapancreatic solid pseudopapillary tumor: case report and review of the literature. Int J Clin Oncol.

[8] Coleman KM, Doherty MC, Bigler SA (2003). Solid-pseudopapillary tumor of the pancreas. Radiographics.

[9] Mortenson MM, Katz MH, Tamm EP (2008). Current diagnosis and management of unusual pancreatic tumors. Am J Surg.

[10] Mao C, Guvendi M, Domenico DR, Kim K, Thomford NR, Howard JM (1995). Papillary cystic and solid tumors of the pancreas: a pancreatic embryonic tumor? Studies of three cases and cumulative review of the world’s literature. Surgery.

[11] Christodoulidis G, Zacharoulis D, Barbanis S, Katsogridakis E, Hatzitheofilou K (2007). Heterotopic pancreas in the stomach: a case report and literature review. World J Gastroenterol.

[12] Fukunaga M (2001). Pseudopapillary solid cystic tumor arising from an extrapancreatic site. Arch Pathol Lab Med.

[13] Hibi T, Ojima H, Sakamoto Y (2006). A solid pseudopapillary tumor arising from the greater omentum followed by multiple metastases with increasing malignant potential. J Gastroenterol.

[14] Kim YI, Kim ST, Lee GK, Choi BI (1990). Papillary cystic tumor of the liver. A case report with ultrastructural observation. Cancer.

[15] Deshpande V, Oliva E, Young RH (2010). Solid pseudopapillary neoplasm of the ovary: a report of 3 primary ovarian tumors resembling those of the pancreas. Am J Surg Pathol.

[16] Cheuk W, Beavon I, Chui DT, Chan JK (2011). Extrapancreatic solid pseudopapillary neoplasm: report of a case of primary ovarian origin and review of the literature. Int J Gynecol Pathol.

[17] Walter T, Hommell-Fontaine J, Hervieu V (2011). Primary malignant solid pseudopapillary tumors of the gastroduodenal area. Clin Res Hepatol Gastroenterol.

[18] Kosmahl M, Seada LS, Jänig U, Harms D, Klöppel G (2000). Solid-pseudopapillary tumor of the pancreas: its origin revisited. Virchows Arch.

[19] Santini D, Poli F, Lega S (2006). Solid-papillary tumors of the pancreas: histopathology. JOP.

[20] Tang LH, Aydin H, Brennan MF, Klimstra DS (2005). Clinically aggressive solid pseudopapillary tumors of the pancreas: a report of two cases with components of undifferentiated carcinoma and a comparative clinicopathologic analysis of 34 conventional cases. Am J Surg Pathol.

[21] Canzonieri V, Berretta M, Buonadonna A (2003). Solid pseudo-papillary tumour of the pancreas. Lancet Oncol.

[22] Nishihara K, Nagoshi M, Tsuneyoshi M, Yamaguchi K, Hayashi I (1993). Papillary cystic tumors of the pancreas. Assessment of their malignant potential. Cancer.

[23] Reddy S, Cameron JL, Scudiere J (2009). Surgical management of solid-pseudopapillary neoplasms of the pancreas (Franz or Hamoudi tumors): a large single-institutional series. J Am Coll Surg.

[24] Maffuz A, Bustamante Fde T, Silva JA, Torres-Vargas S (2005). Preoperative gemcitabine for unresectable, solid pseudopapillary tumour of the pancreas. Lancet Oncol.

[25] Hah JO, Park WK, Lee NH, Choi JH (2007). Preoperative chemotherapy and intraoperative radiofrequency ablation for unresectable solid pseudopapillary tumor of the pancreas. J Pediatr Hematol Oncol.

